# Deciphering immune cell interaction aberrations in necrotizing enterocolitis: the regulatory role of DHRS7

**DOI:** 10.3389/fimmu.2025.1743878

**Published:** 2026-01-06

**Authors:** Kexin Gao, Yuehua Chen, Jingjing Lu, Wenliang Ge, Qiyou Yin

**Affiliations:** 1 Pediatric Surgery, Affiliated Hospital of Nantong University, Nantong, Jiangsu, China; 2 Pediatric Surgery, Medical School of Nantong University, Nantong, Jiangsu, China; 3 Clinical Medical Research Center, Affiliated Hospital of Nantong University, Nantong, Jiangsu, China

**Keywords:** candidate drugs, DHRS7, immune infiltration, inflammation, necrotizing enterocolitis

## Abstract

**Background:**

Necrotizing enterocolitis (NEC) is a severe inflammatory condition predominantly affecting preterm infants and is one of the leading causes of neonatal mortality worldwide. Despite its significant impact, the pathogenesis of NEC remains unclear, particularly regarding the role of aberrant immune cell responses and uncontrolled inflammation. This study aimed to investigate how dehydrogenase/reductase 7 (DHRS7), a protein potentially influencing immune cell function and inflammatory signaling pathways, contributes to the development of NEC.

**Methods:**

Screening and analyzing differential proteins between normal samples and NEC samples through proteomic sequencing. Bioinformatics analyses of the GSE46619 dataset were performed, including differential expression profiling, receiver operating characteristic (ROC) curve analysis for diagnostic efficacy, gene set enrichment analysis (GSEA) for pathway enrichment, and cell-type identification by estimating relative subsets of RNA transcripts (CIBERSORT) to evaluate immune cell infiltration. Gene interactions and functional pathways were further explored using gene multiple association network integration algorithm (GeneMANIA), KEGG and GO analyses. Molecular docking studies were conducted to investigate the binding interactions of DHRS7 with small molecules. A murine model of NEC was established to evaluate inflammatory responses through Hematoxylin and eosin (H&E) staining and immunofluorescence, as well as to assess DHRS7 expression using immunohistochemistry. Additionally, lipopolysaccharide (LPS)-stimulated rat IEC-6 cells served as an *in vitro* model, with DHRS7 expression levels and inflammatory cytokines quantified using RT-qPCR and western blot.

**Results:**

Proteomic sequencing and bioinformatics analysis revealed that DHRS7 was significantly downregulated in NEC tissues and could serve as a diagnostic marker. GSEA enrichment analysis indicated a strong association between DHRS7 and inflammatory signaling pathways. Moreover, immune profiling showed an increase in neutrophil and macrophage infiltration in tissues with low DHRS7 expression. DHRS7 demonstrated a high affinity for mitogen-activated protein kinase (MEK) inhibitor small molecules. Additionally, DHRS7 expression was markedly reduced in both NEC mouse models and LPS-induced IEC-6 cells.

**Conclusion:**

DHRS7 is significantly downregulated in NEC tissues, potentially serving as a diagnostic marker, while its decreased expression is associated with increased neutrophil and macrophage infiltration, underscoring its vital role in immune modulation in NEC.

## Introduction

Necrotizing enterocolitis (NEC) is a severe inflammatory gastrointestinal disorder predominantly affecting premature infants, representing a significant challenge in neonatal care (1). Despite advancements in treatment strategies, the prevalence of NEC in this vulnerable population remains high, with alarmingly high mortality rates estimated to range from 20% to 30% (2–5). The pathophysiology of NEC remains insufficiently understood. Current thinking indicates that dysregulation of the inflammatory immune response may play a key role in the pathogenesis of NEC (6). Innate immune cells, including neutrophils and macrophages, play an essential role in the inflammatory and immune responses to pathogens and infections. Inflammatory macrophage infiltration has been observed in the intestinal tissues of NEC patients as well as in the NEC mouse model (7–9). In clinical practice, differentiating NEC from other common conditions in preterm infants can be quite challenging during the early stages of the disease, underscoring the critical need for identifying specific biomarkers.

Steroids are a class of organic compounds characterized by a distinctive structure consisting of four fused carbon rings (10). They play essential roles in various physiological functions within the body, including the regulation of metabolism, immune response, and inflammation (11). The identification of sex steroids and glucocorticoids (GC) has significantly promoted the clinical utilization of steroid therapies (12). Recent studies have demonstrated that endogenous steroids are crucial regulators of the immune system (13). Furthermore, the non-calcemic effects of vitamin D on immune cells, particularly T cells, underscore its potential in treating various allergic conditions (14).

Enzymes in the short-chain dehydrogenase/reductase (SDR) superfamily are integral to the metabolism of steroids and retinoids, with dehydrogenase/reductase member 7 (DHRS7, SDR34C1) isoform 1 being particularly noteworthy. DHRS7 integrates NADP+/NADPH redox sensing and inflammatory lipid signaling. This integration occurs through the Oxoeicosanoid pathway (15, 16). Additionally, DHRS7 has emerged as an immune-related prognostic biomarker for both kidney renal clear cell carcinoma (KIRC) and pan-cancer (17). Despite its potential significance, research on the specific role of DHRS7 in inflammation and immune response is still severely restricted.

In this study, we conducted a novel investigation into the role of DHRS7 in NEC. Utilizing bioinformatics and proteomic sequencing analyses, we found that DHRS7 is significantly downregulated in NEC tissues, demonstrating a close association with inflammatory signaling pathways. Notably, in tissues where DHRS7 expression was diminished, there was an increase in the infiltration of neutrophils and macrophages. Furthermore, DHRS7 exhibited a strong affinity for mitogen-activated protein kinase (MEK) inhibitors, highlighting its potential as a diagnostic marker for NEC. Additionally, our results indicated that in NEC animal models, the expression of DHRS7 was significantly lower compared to the normal group. Similarly, DHRS7 expression was markedly reduced in LPS-induced IEC-6 cells. These findings suggest that DHRS7 may play a critical role in the pathogenesis and progression of NEC, offering new avenues for future research.

## Materials and methods

### Identification of DHRS7 in NEC and gene set enrichment analysis

Differential expression analysis of the GSE46619 dataset, which included 9 control samples and 11 NEC samples, was performed using the “limma” package in R. Expression levels of DHRS7 were visualized using boxplots. The diagnostic performance was assessed through receiver operating characteristic (ROC) curve analysis, employing the “pROC” package. GSEA for DHRS7 was carried out using GSEA software (version 4.3.2), with 1,000 permutations; pathways with a false discovery rate (FDR) less than 0.05 were considered significantly enriched. The data were normalized to reads per kilobase per million mapped reads (RPKM). Differentially expressed genes (DEGs) were identified by |log_2_FC| > 1 and a *P*-value < 0.05. All analyses adhered to the MIQE guidelines.

### Immune infiltration and gene network analysis

Immune cell fractions for 22 subsets within the GSE46619 dataset were quantified using cell-type identification by estimating relative subsets of RNA transcripts (CIBERSORT). The relationship between DHRS7 expression and immune cell proportions was evaluated through Pearson correlation analysis. To construct functional protein-protein interaction networks for DHRS7, gene multiple association network integration algorithm (GeneMANIA) was utilized, allowing for the identification of co-expressed genes, physical interactions, and pathway partners. Enrichment analyses of the resulting gene networks for KEGG pathways and GO terms were conducted using the “clusterProfiler” package in R.

### Molecular docking analysis

The GSE46619 cohort was stratified into high- and low-DHRS7 expression groups based on median expression levels. Significantly upregulated DEGs (|log_2_FC| > 1, FDR < 0.05) in the high-expression group were submitted to the Connectivity Map (CMap) database (https://clue.io) to identify potential compounds targeting DHRS7. The protein structures of the hub genes were extracted from the AlphaFold Protein Structure Database (https://alphafold.ebi.ac.uk), while the structural data for small-molecule compounds were obtained in SDF format from PubChem (https://pubchem.ncbi.nlm.nih.gov/) and subsequently converted to PDB format using OpenBabel software (version 2.4.1). We then utilized the CB-Dock2 database (https://cadd.labshare.cn/cb-dock2/php/index.php) for molecular docking, employing the surface curvature-based cavity detection method (CurPocket) to identify potential binding regions within the protein (18). Blind docking was performed on these identified potential binding sites of the target protein, followed by the application of AutoDock Vina to automatically detect potential binding sites and estimate the binding free energy (ΔG, kcal/mol) using an empirical scoring function. Compounds demonstrating strong binding affinity, with ΔG < -7 kcal/mol, were successfully screened. The protein-ligand interactions were then visualized using PyMOL and Discovery Studio 2021, providing further insights into the molecular interactions.

### Patients, sample collection

Tissue samples from intestinal segments were obtained from six patients with NEC and six patients with intestinal atresia at the Affiliated Hospital of Nantong University. All *in vitro* clinical samples were stored at -80 °C. This study complied with all relevant ethical regulations and was approved by the Ethics Committee of Nantong University Hospital (Approval No. 2025-L031). And all participants provided written informed consent to participate in this study.

### Proteomics sequencing analysis

The Proteomic data analysis was performed by Shanghai OE Biotech Co., Ltd. (Shanghai, China). Approximately 100 mg frozen samples were ground in liquid nitrogen, homogenized with phenol buffer, mixed with phenol, and incubated at 4 °C. The phenolic phase was centrifuged, combined with ammonium acetate-methanol, and precipitated at -20 °C overnight. After processing, protein in the supernatant was quantified by BCA assay. For SDS-PAGE, 10 μg protein was analyzed post-staining; for digestion, 50 μg protein was digested with trypsin (at a protein - to - trypsin ratio of 50:1) at 37 °C overnight. Peptides were desalted, dried, resuspended, mixed with iRT peptides, and analyzed by LC-MS/MS (Nanoelute2-timsTOF HT, 10-min gradient). Raw data were imported into Proteoscape with a custom database, ensuring that the rate of peptide false positives was less than 1%.

### NEC models

Animal experiments were approved by the Institutional Animal Care and Use Committee of Nantong University (Approval No. P20250315-004) and were carried out in compliance with institutional guidelines. Preterm C57BL/6 mice (Jingqi Biotechnology Co. Ltd., Nantong, China) were randomly allocated to either the NEC group (n = 30) or the dam-fed control group (n = 30). The NEC model was induced as previously described with modifications: the pups experienced twice-daily cycles of hypoxia (99.9% N_2_, 120 s) and cold stress (4 °C, 10 min) and received gavage feedings (30% Esbilac formula, 50 µL) every 4 h for a total of 96 h. In contrast, control pups remained with their dams throughout the experiment. Terminal ileum samples were collected 4 days post-partum, fixed in 4% paraformaldehyde (Servicebio, Wuhan, China), or snap-frozen for subsequent analyses. Body weight was monitored daily to assess the health status of the pups.

### Hematoxylin and eosin staining

Ileum samples were collected and fixed in 4% paraformaldehyde for 24 h to 48 h. The specimens were then paraffin-embedded and sectioned into 5 μm thick slices. Following sectioning, the samples were stained with H&E stainning. Histopathological evaluations were performed using an Olympus light microscope (Tokyo, Japan), with the severity of intestinal injury scored in a blinded manner based on established criteria. The scoring scheme was as follows: 0 (normal), 1 (epithelial sloughing), 2 (partial villus necrosis with moderate submucosal separation), 3 (extensive villus necrosis with severe submucosal separation), and 4 (transmural necrosis). A score of ≥ 2 was designated as indicative of NEC, with confirmation provided by two independent pathologists blinded to the sample identities.

### Immunohistochemistry

Formalin-fixed and paraffin-embedded tissue sections were deparaffinized using xylene and then rehydrated through a series of graded ethanol solutions. For antigen retrieval, sections were subjected to treated with 0.01 M citrate buffer (pH 6.0) using microwave heating. Following antigen retrieval, the sections were blocked with 3% (v/v) goat serum (Servicebio, Wuhan, China) for 1 h at room temperature. The samples were then incubated overnight at 4 °C with a rabbit DHRS7 primary antibody (1:2000, Proteintech, Wuhan, China). Immunoreactivity was visualized using 3,3’-diaminobenzidine (DAB; Servicebio, Wuhan, China), followed by counterstaining with hematoxylin. After staining, the sections were dehydrated, cleared in xylene, and examined under an Olympus BX53 light microscope (Tokyo, Japan). The staining intensity was classified as either positive, indicating moderate to strong staining, or negative, representing weak or no staining, based on the reactivity of the IHC marker.

### Cell culture and inflammatory challenge

Rat intestinal epithelial (IEC-6) cells (ZM0783; Zhong Qiao Xin Zhou Biotechnology, Shanghai, China) were cultured in high-glucose DMEM (Gibco, USA) supplemented with 10% fetal bovine serum (Sigma-Aldrich, USA) and 1% penicillin/streptomycin (NCM Biotech, Suzhou, China) at 37°C in a humidified atmosphere containing 5% CO_2_. To induce an inflammatory response, cells were treated with 10 µg/mL LPS (L2630, Sigma-Aldrich, USA) for durations ranging from 0 h to 24 h.

### RNA Extraction and RT-qPCR

Total RNA was extracted from intestinal tissues or IEC-6 cells using TRIzol reagent (Thermo Fisher, USA) in accordance with the manufacturer’s instructions. The purity and concentration of the RNA were assessed spectrophotometrically using a NanoDrop (Thermo Scientific, USA), ensuring an A260/A280 ratio greater than 1.8. For complementary DNA (cDNA) synthesis, 1 μg of the isolated RNA was reverse-transcribed using the cDNA One Strand Synthesis Kit (Vazyme, Nanjing, China) under standard conditions. RT-qPCR was conducted using SYBR qPCR SuperMix (Vazyme, Nanjing, China). Relative mRNA expression levels were calculated using the 2^−ΔΔCt^ method, with normalization to β-actin. The sequences of the forward (F) and reverse (R) primers are provided below: DHRS7 (*Mus musculus*)-F: 5’-GGAGCGTCAAGTGGCATTG -3’, R: 5’-AAGGGGCAGAACCAGTATATCT-3’; β-actin (*Mus musculus*): F: 5’-GTGACGTTGACATCCGTAAAGA-3’, R: 5’-GCCGGACTCATCGTACTCC-3’; DHRS7 (*Rattus norvegicus*): F: 5’-GTGCAGTTCCTACGCTTCCT-3’, R: 5’-CTCACCAATGCCGCTTGATA-3’; IL-6 (*Rattus norvegicus*): F: 5’-AGAGACTTCCAGCCAGTTGC-3’, R: 5’-AGCCTCCGACTTGTGAAGTG -3’, TNF-α (*Rattus norvegicus*):F: 5’-CTGTGCCTCAGCCTCTTCTC-3’, R: 5’-ACTGATGAGAGGGAGCCCAT-3’, β-actin (*Rattus norvegicus*): F: 5’-TGCTGACAGGATGCAGAAGG-3’, R: 5’- CGGACTCATCGTACTCCTGC-3’.

### Western blot

IEC-6 cells were lysed in RIPA buffer (Beyotime, Shanghai, China) supplemented with protease inhibitors. Protein concentrations were determined using a BCA assay kit (Vazyme, Nanjing, China). Proteins were separated on 10% SDS-PAGE gels (Yeasen, Shanghai, China) and subsequently transferred onto PVDF membranes (Millipore, Billerica, USA). Membranes were blocked with 5% (w/v) skim milk (Sigma-Aldrich, USA) for 2 h at room temperature before being incubated overnight at 4 °C with the following primary antibodies: DHRS7 antibody (1:1000, Proteintech, Wuhan, China) and β-actin antibody (1:5000, Proteintech, Wuhan, China). After three washes with TBS-T (Servicebio, Wuhan, China), membranes were incubated with HRP-conjugated secondary antibodies (1:8000, Beyotime, Shanghai, China) for 2 h at room temperature. Protein bands were detected using a chemiluminescent HRP substrate (NCM Biotech, Suzhou, China) and visualized using a Tanon imaging system (Shanghai, China). Band intensities were quantified with ImageJ software and normalized to β-actin.

### Immunofluorescence

Antigen-retrieved intestinal tissues were permeabilized with 0.1% Triton X-100 and subsequently blocked with 5% (w/v) bovine serum albumin (BSA; Sigma-Aldrich) for 1 h at room temperature. The samples were incubated overnight at 4°C with primary antibodies against Claudin-1 and ZO-1 (both 1:1000, Proteintech, Wuhan, China). Following three washes with PBS, samples were incubated with fluorophore-conjugated secondary antibodies (1:500, Invitrogen) for 1 h in the dark. Nuclei were stained with DAPI (5 μg/mL, Sigma-Aldrich) for 5 min. Images were acquired using a Zeiss LSM 880 confocal microscope (Oberkochen, Germany).

### Statistical analyses

Data are presented as mean ± standard error of the mean (SEM). Group comparisons were conducted using Student’s t-test for two groups or one-way analysis of variance (ANOVA) for three or more groups, followed by Tukey’s *post-hoc* test for multiple comparisons. ROC curve analysis was performed to evaluate diagnostic accuracy. Correlation analyses were carried out using Pearson’s test for parametric data and Spearman’s test for non-parametric data. All animal and cellular experiments were independently replicated three times. Statistical significance was defined as *P* < 0.05.

## Results

### Low expression of DHRS7 was closely associated with inflammatory pathways in NEC tissues

**Figure 1:**
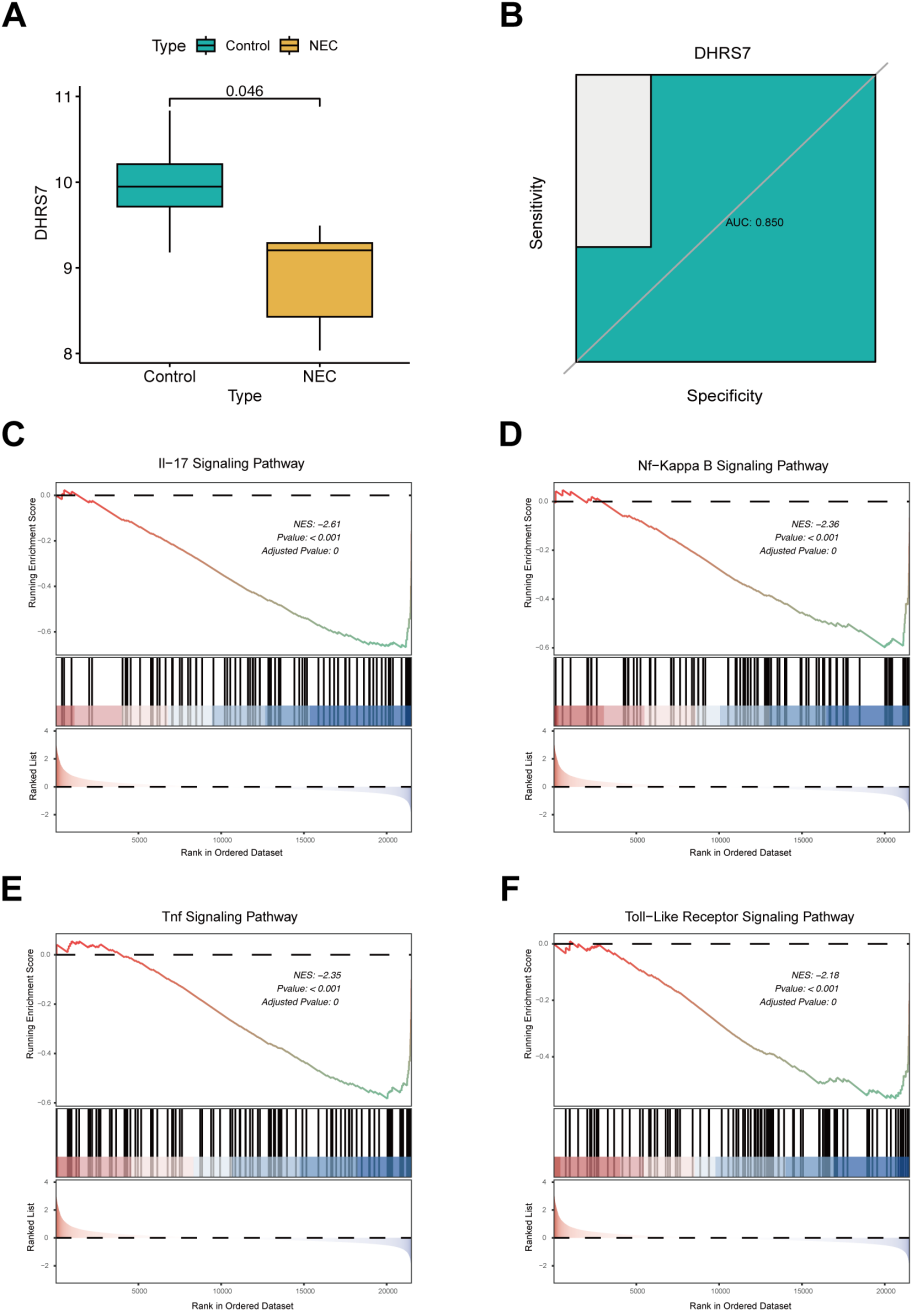
Diagnostic evaluation and pathway enrichment analysis of DHRS7. **(A)** Differential expression analysis of DHRS7 in GSE46619. **(B)** ROC curve analysis of DHRS7 expression in NEC and control groups. **(C–F)** GSEA analysis of DHRS7.

Differential expression analysis of the GSE46619 dataset revealed a significant downregulation of DHRS7 in NEC samples compared to controls (**Figure 1A**). ROC curve analysis further substantiated its diagnostic potential, yielding an area under the curve (AUC) of 0.89, with a 95% confidence interval (CI) ranging from 0.82 to 0.96 (**Figure 1B**). GSEA indicated that the downregulation of DHRS7 was significantly associated with various inflammatory pathways, including the IL-17 signaling pathway, NF-κB signaling cascade, Toll-like receptor signaling, and TNF signaling pathway (**Figures 1C–F**). These findings suggest that DHRS7 may play a crucial role in modulating pro-inflammatory cytokine networks.

### DHRS7 played a key role in NEC pathogenesis through metabolic reprogramming and mTOR activation.

**Figure 2:**
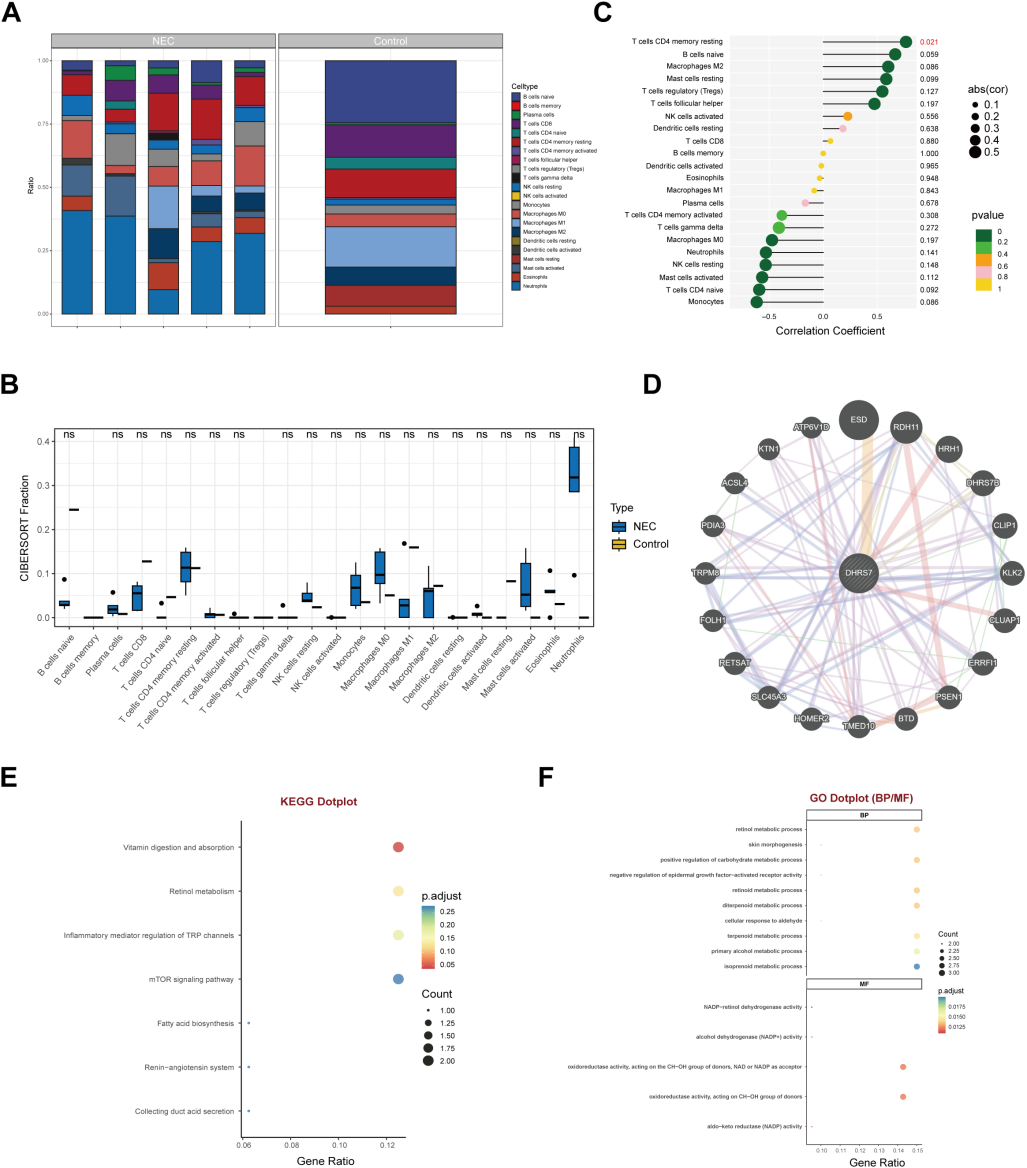
Immune infiltration profiling and functional gene networks associated with DHRS7 in NEC. **(A)** Bar charts of 22 types of immune cells in NEC and control groups. NEC (n=5); Control (n=1). **(B)** Comparative Immunophenotyping of immune cell subsets in NEC and control groups via CIBERSORT. **(C)** DHRS7 is associated with multiple immune cells. **(D)** GeneMANIA-driven co-expression Network analysis identifies DHRS7 as a central hub gene with implications in metabolic dysregulation pathways. **(E, F)** KEGG **(E)** and GO **(F)** enrichment dotplot analysis reveals dysregulated metabolic pathways and molecular functions.

To explore the role of DHRS7 in the inflammatory immune response, we conducted an immune infiltration analysis. This analysis revealed profound alterations in the distribution of 22 immune cell types compared with the control group, including an increase in the proportion of naive B cells and activated mast cells, coupled with a decrease in the proportion of CD4^+^ naive T cells (**Figures 2A, B**). Notably, we found a positive correlation between DHRS7 expression and CD4^+^ T cell infiltration (**Figure 2C**). GeneMANIA co-expression networks identified DHRS7 as a crucial hub that connects metabolic enzymes (such as HSD17B2 and AKR1C3) with oncogenic regulators, including TP53 and mTOR. Additionally, KEGG/GO enrichment analyses underscored dysregulated pathways encompassing fatty acid biosynthesis, retinol metabolism, and oxidoreductase activity (**Figures 2D–F**). Collectively, these findings suggest that metabolic reprogramming mediated by DHRS7 plays a significant role in the pathogenesis of NEC, linking mTOR pathway activation to the remodeling of the immune microenvironment.

### Identification of candidate drugs and molecular docking via differential expression analysis

**Figure 3:**
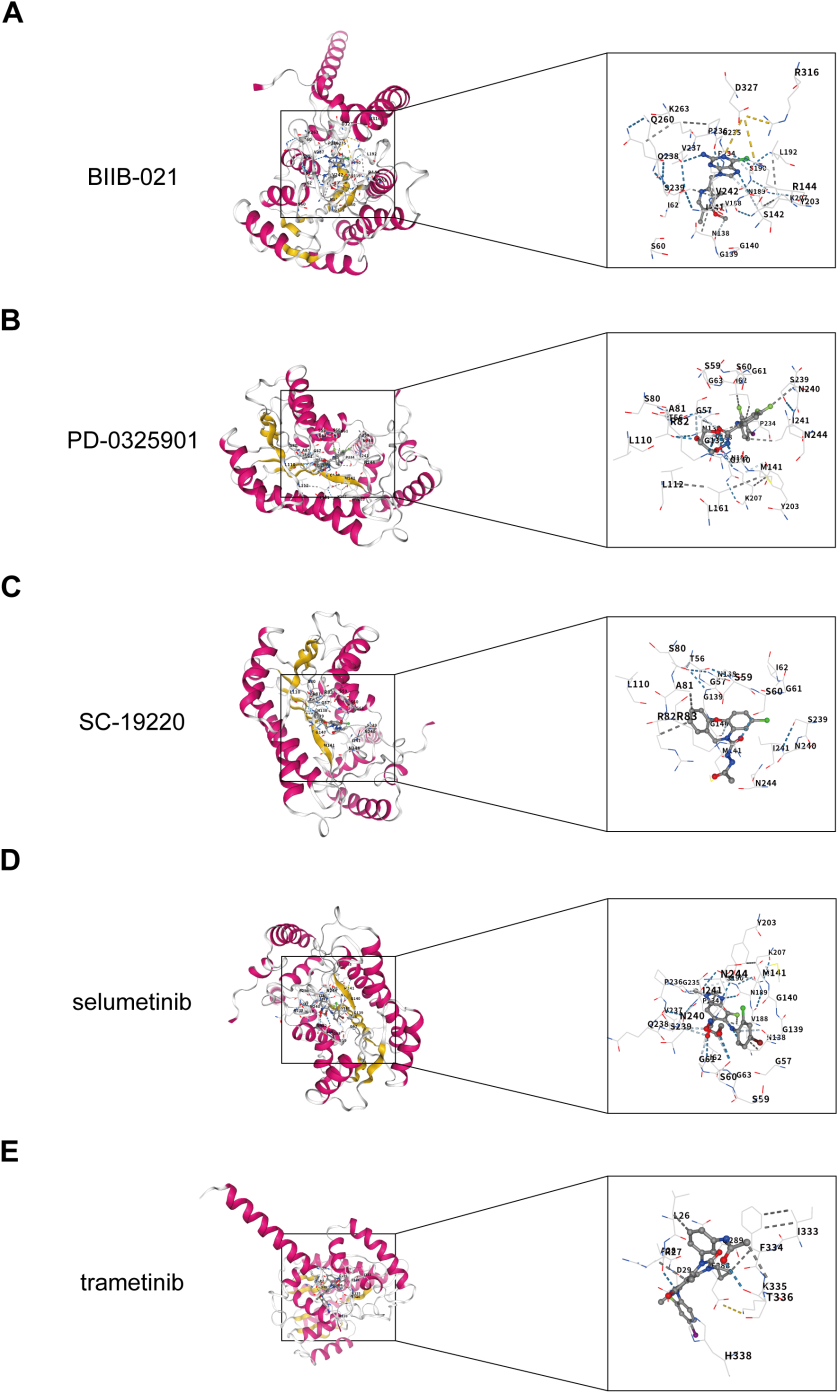
Molecular docking analysis of key genes and active compounds in NEC. **(A–E)** Molecular docking results showing the binding between the key gene DHRS7 and the active compound of BIIB-02 **(A)**, PD-0325901 **(B)**, SC-19220 **(C)**, selumetinib **(D)**, trametinib **(E)**.

The GSE46619 dataset was stratified into low- and high-expression cohorts based on the median expression level of DHRS7. This stratification led to the identification of 86 significantly upregulated DEGs in the high-expression group (|log_2_FC| > 1, FDR < 0.05). Connectivity Map (CMAP) analysis identified five candidate drugs-BIIB-02, PD-0325901, SC-19220, selumetinib, and trametinib-based on negative enrichment scores. Molecular docking simulations demonstrated that trametinib (ΔG = -8.5 kcal/mol) and BIIB-02 (ΔG = -8.4 kcal/mol) emerged as the most promising candidates, both classified as “excellent” due to their ΔG values being less than -7. Additionally, PD-0325901 (ΔG = -7.9 kcal/mol), SC-19220 (ΔG = -7.4 kcal/mol), and selumetinib (ΔG = -7.3 kcal/mol) also met the stability criteria (**Figures 3A–E**).

### Proteomic sequencing-based validation of DHRS7 expression

**Figure 4:**
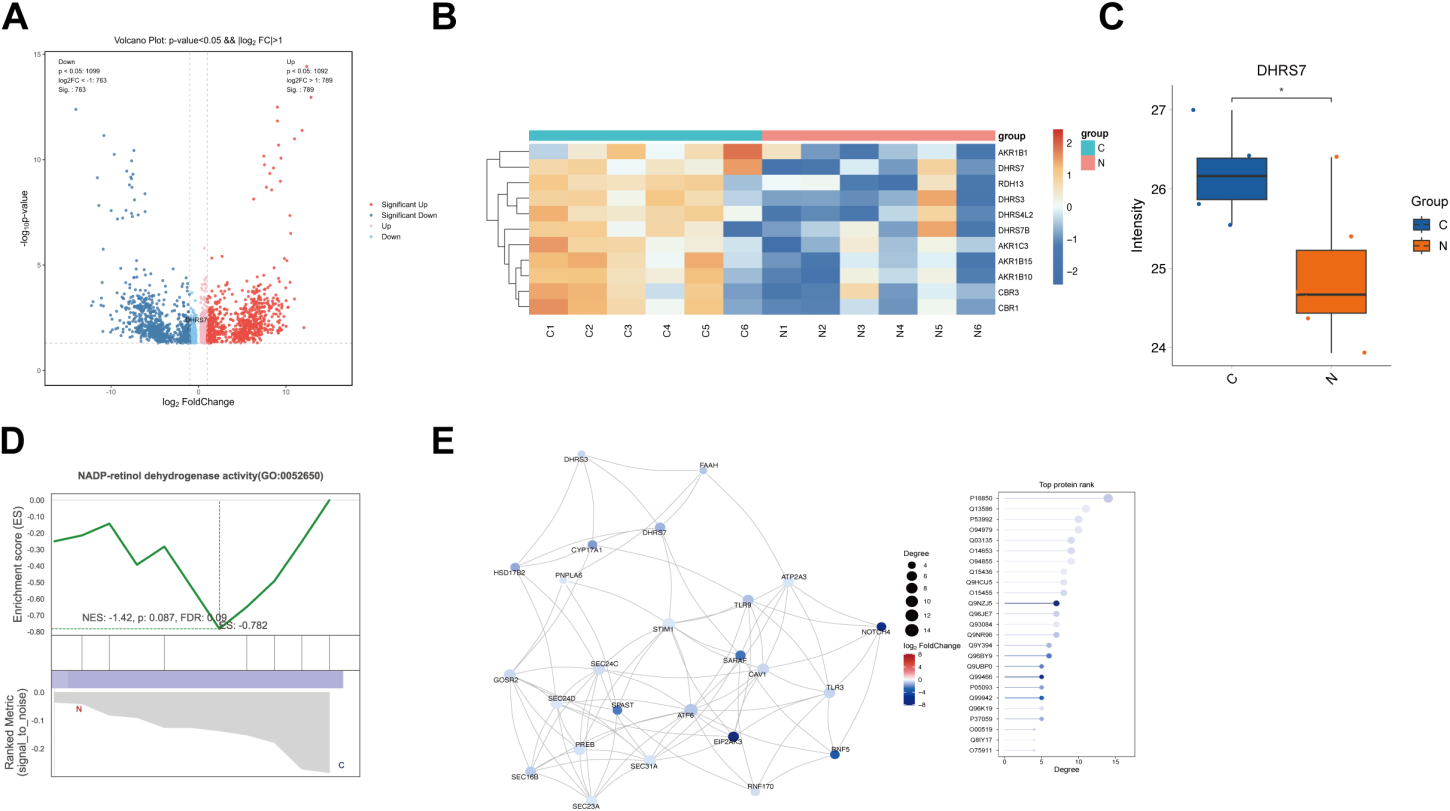
Expression validation of DHRS7 in proteomic sequencing. **(A)** Volcano plot for the differential expression analysis, in which red dots indicate upregulated differentially expressed proteins and blue dots indicate downregulated differentially expressed proteins in NEC. **(B)** Heatmap showing the increased and decreased expressions of the DHRS7-related genes. **(C)** DHRS7 expression bar chart in the proteomics sequencing database. **(D)** GSEA analysis of DHRS7. **(E)** Visualization of the protein-protein interaction (PPI) network for DEPs associated with endoplasmic reticulum (ER) membrane function was constructed using STRING and Cytoscape.

In proteomics sequencing, a total of 1552 differentially expressed proteins were identified, including 789 upregulated genes and 763 downregulated genes. Among these, DHRS7 exhibited significantly reduced expression in NEC (|log_2_FC| > 1, *P*-value < 0.05; **Figures 4A–C**). GSEA analysis indicated that DHRS7 downregulation was closely associated with NADP-retinol dehydrogenase activity (**Figure 4D**). Co-expression network analysis identified robust interactions between DHRS7 and oxidoreductase family members and immune-related proteins, suggesting its involvement in metabolic and inflammatory pathways (**Figure 4E**). These findings collectively suggest DHRS7 may play a dual role in redox regulation and disease progression.

### Reduced DHRS7 expression was associated with inflammatory and structural damage in NEC

**Figure 5:**
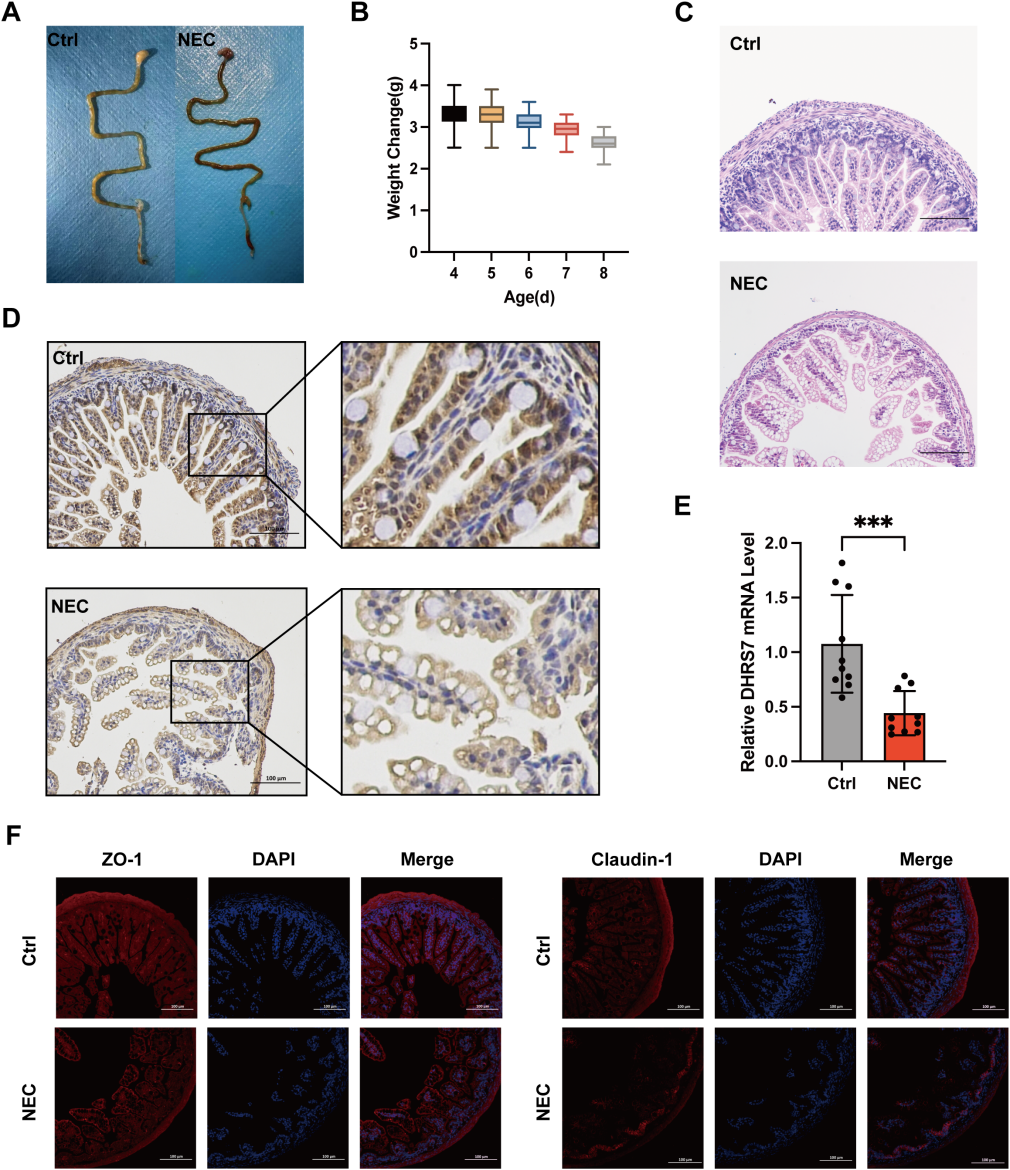
Validation of the expression of DHRS7 in animal model of NEC. **(A)** Comparative gross morphology of intestinal tissues between NEC and control group. **(B)** Body weight changes in NEC Mice. **(C)** H&E staining of intestinal tissues in control and NEC model mice. **(D)** Representative images of DHRS7 immunohistochemistry in NEC and control tissue. **(E)** DHRS7 mRNA level in control and NEC mice. **(F)** Immunofluorescence staining of intestinal barrier proteins (ZO-1 and Claudin-1) in NEC and control mice. ***P < 0.001.

To further explore the role of DHRS7 in NEC, we induced NEC in C57BL/6 mice through a combination of hypoxia/hypothermia and formula feeding. The NEC mice exhibited pronounced intestinal pathological alterations, including intraluminal hemorrhage and intestinal emphysema (**Figure 5A**), as well as significant growth impairment (*P* < 0.01 compared to the control group on day 7; **Figure 5B**). H&E staining confirmed the destruction of villi and notable inflammatory infiltration (**Figure 5C**). Immunofluorescence assays revealed a significant reduction in the expression levels of ZO-1 and Claudin-1 within the intestinal barrier of the NEC mice (**Figure 5F**). Additionally, RT-qPCR revealed that the DHRS7 mRNA expression in NEC tissues was significantly decreased compared to the control group (**Figure 5E**). Consistently, immunohistochemical analysis demonstrated a marked reduction in DHRS7 protein expression in NEC tissues (**Figure 5D**). Collectively, these results underscore the pivotal role of DHRS7 in the pathological progression of NEC.

### LPS treatment enhanced inflammatory responses and reduced DHRS7 expression in IEC-6 cells

**Figure 6:**
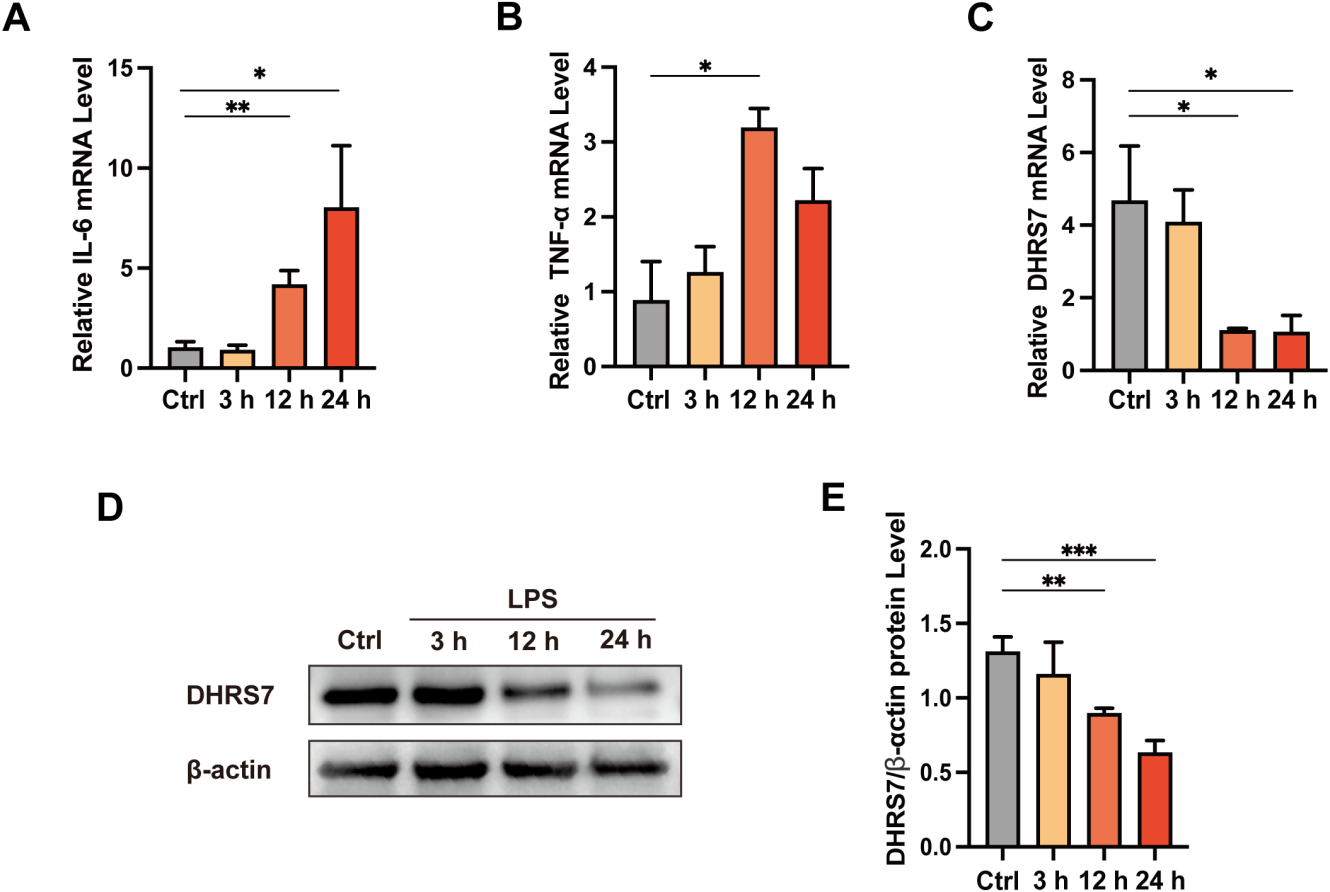
Validation of DHRS7 and inflammatory cytokine expression in LPS-stimulated IEC-6 Cells. **(A, B)** The mRNA levels of IL-6, TNF-α detected by RT-qPCR. **(C)** Relative mRNA expression of DHRS7 measured by RT-qPCR in IEC-6 cells treated with LPS (10 μg/mL) for the indicated times. **(D, E)** Western blot analysis of DHRS7 protein. *P < 0.05, **P < 0.01, ***P < 0.001.

We further treated IEC-6 cells with LPS to establish an inflammation model. First, we confirmed the successful induction of the inflammation model by observing a significant increase in inflammatory markers. IL-6 peaked at 24 h and TNF-α reached its highest levels at 12 h (**Figures 6A, B**). Subsequently, we measured the expression of DHRS7 and found a time-dependent downregulation of its expression (**Figure 6C**). Additionally, Western blot analysis confirmed a substantial decrease in DHRS7 protein levels following LPS treatment (**Figures 6D, E**). These findings provide compelling evidence supporting the negative correlation between DHRS7 expression and inflammatory responses.

## Discussion

NEC is a severe inflammatory intestinal disorder that predominantly affects neonates. Despite recent research advancements, its underlying causes remain inadequately understood (19, 20). The onset of NEC is influenced by a complex multiple of factors, particularly inappropriate innate immune responses, which, in conjunction with damage to the intestinal barrier, initiate an inflammatory cascade (6). Diagnosis is mainly based on non-specific clinical signs, such as abdominal distension and hematochezia, alongside radiological findings like intestinal pneumatosis (5, 21). This often results in delays in treatment, with approximately 50% of cases requiring surgical resection (22). Current treatment focuses on supportive care and antibiotics, underscoring an urgent need for mechanism-based biomarkers and targeted therapies to improve outcomes for affected infants (23).

Undocumented SDRs may play pivotal physiological and pathological roles in various diseases, including NEC. Investigating their functions is expected to enhance our understanding of disease mechanisms, which is essential for developing innovative diagnostic and therapeutic approaches. DHRS7, an NADPH-dependent oxidoreductase belonging to the SDR superfamily, is recognized for its catalytic activity in reducing carbonyl groups in steroid hormones and retinoids (15–17). However, it is considered an “orphan” SDR due to the limited understanding of its physiological roles. In KIRC and pan-cancer studies, DHRS7 has emerged as an immune-related prognostic biomarker (17). It also demonstrates a protective role in prostate cancer, with its expression positively correlated with patient survival rates, and holds potential as a tumor suppressor (24). In our study, we initially identified a significant downregulation of DHRS7 in NEC tissues through proteomic sequencing and bioinformatics analysis. This finding was subsequently validated using NEC mouse models and an LPS-induced IEC-6 cell model. Our research reveals a potential association between DHRS7 and NEC, offering new insights into this area of study.

Current research on DHRS7 is limited, leaving its role in NEC largely unexplored. Our proteomic sequencing and bioinformatics analysis revealed a significant association between DHRS7 and inflammatory signaling pathways. Furthermore, tissues with low expression of DHRS7 demonstrated a marked increase in the infiltration of neutrophils and macrophages. The metabolism of immune cells undergoes metabolic reprogramming based on the energy demands of activation, differentiation, and effector functions, influenced by steroid hormones such as glucocorticoids, androgens, progestogens, and estrogens. These hormones regulate intracellular metabolic pathways, including glycolysis, the tricarboxylic acid cycle, and oxidative phosphorylation, through both genomic and non-genomic mechanisms, thereby impacting immune function and inflammatory responses. In future research, we will delve into the regulatory role of DHRS7 in inflammation and immune metabolism.

Importantly, DHRS7 showed a strong affinity for small molecule MEK inhibitors. It integrates NADP+/NADPH redox sensing with inflammatory lipid signaling via the Oxoeicosanoid pathway (25). These findings underscore the potential impact of DHRS7 on immune regulation in NEC, suggesting that it could be a promising target for therapeutic intervention to alleviate inflammation in related conditions. However, the current proposal identifying DHRS7 as a therapeutic target for NEC remains underexplored in terms of its translational potential. To address this critical gap, future investigations should prioritize structure-based drug discovery, utilizing AlphaFold-predicted protein models to design selective agonists or antagonists targeting DHRS7’s catalytic domain or allosteric regulatory sites. Future research is required to further elucidate the mechanisms by which DHRS7 influences these pathways and to assess its viability as a biomarker or therapeutic target in inflammatory diseases.

## Conclusion

In conclusion, our integrated analysis of proteomic sequencing and bioinformatics revealed significant downregulation of DHRS7 in NEC tissues, positioning it as a promising diagnostic biomarker. This reduction in DHRS7 expression was corroborated by findings in NEC mouse models and LPS-induced IEC-6 cells. Additionally, GSEA enrichment analysis linked DHRS7 to key inflammatory signaling pathways. Notably, lower DHRS7 levels were associated with increased neutrophil and macrophage infiltration, highlighting its role in immune modulation. Moreover, DHRS7 demonstrated a high affinity for small molecule MEK inhibitors. Together, these findings underscore DHRS7’s potential as a therapeutic target and biomarker for NEC.

## Data Availability

The datasets presented in this study can be found in online repositories. The names of the repository/repositories and accession number(s) can be found in the article/supplementary material.
